# Harmonizing culture and consumer psychology: optimizing color schemes for children’s product design inspired by traditional ornaments

**DOI:** 10.1186/s40359-024-01644-6

**Published:** 2024-03-18

**Authors:** Ling Lin Liang, Nazik Hangeldiyeva

**Affiliations:** School of Art and Design, Zhejiang Sci-Tech University, Hangzhou, China

**Keywords:** National ornaments, Traditional patterns, Consumer psychological perception, Traditional color inheritance, Color and color scheme generation, Sustainable children’s product

## Abstract

Color is one of the vital components of product design and the choice of color combination is a critical factor that affects consumer reaction and purchase decisions. However, the impact of traditional colors on color extraction and product design has seldom been studied. The purpose of this study is to discover suitable colors from traditional patterns to create color schemes that will be used in the design of children’s products. Colors were extracted from five major traditional ornaments obtained from Turkmenistan’s national carpets, each representing one of the main Turkmen tribes, using tools like Photoshop, Adobe Color, and EasyRGB, with the objective of investigating psychological-emotional attachment, reaction, and attitudes towards the colors obtained. This initial extraction provided 124 color data points. Initially, extracted colors were refined and used to develop novel color schemes by two independent survey studies. The first survey, based on a sample of 104 parents of preschool children, identified colors that have a positive emotional effect on consumer preferences and provided the basis to develop color schemes. As a result, 25 colors were identified for use in developing 14 color schemes. The second survey, based on a sample of 48 parents of preschool children, identified which color schemes have an attraction for consumers. The survey indicated that 11 out of these 14 schemes were highly favored by the respondents. Based on these results, this study proposed new color schemes for children’s products, extracted from traditional patterns of Turkmenistan and aligned with the psychological perception of the consumers.

**Supplementary Information:**

The online version contains supplementary material available at 10.1186/s40359-024-01644-6.

## Introduction

Traditional elements and patterns remain a valuable part of the national culture of many countries and regions for centuries, which represent a unique symbol of a nation across the world. These traditional elements are full of uniquely identifiable traditional color schemes that are narrowly connected to the individual’s psychological feelings [1]. According to early research, designs carrying colors from cultural elements can evoke emotional responses that affect consumer behavior [2–4]. Consequently, a product design, consisting of traditional color schemes can facilitate increasing the demonstration of national culture to attract customers and motivate them to purchase a product. Indeed, product design that uses culture-inspired elements remains preferable among consumers [1], since it increases the desire of users to brag about national pride through aesthetic, judicious traditional elements [5].

With the rapid development of science and technology, consumer consumption and interest in children’s products generate increased competition and challenge between designers. When consumers purchase a product, they distinguish the product’s basic characteristics and originality according to their own preferences and priorities. Since color has a visual impact on individuals [6] and captures 80% of the whole product impressions [1], designers focus on generating more unique color schemes to attract consumers [7]. Indeed, color has a considerable impact on people’s emotions and perceptions [8], in particular, on children. For instance, Boyatzis and Varghese [9] argued that children demonstrated apparent emotional association with colors, but with gender differences, where gender played a role in distinguishing negative or positive color emotions concerning children’s gender (i.e., boys reacted more positively to the dark colors than girls). However, their study discovered that the link between color and emotions depends on a child’s personal experienced emotions regarding a particular color, as well as, the cognitive development of the child. Consequently, color combinations can affect a consumer’s intentions and purchase decisions, as well as, an attitude toward a product [10]. Nonetheless, designers often struggle to determine the color combinations, and they are unable to assess consumers’ responses unless the product is launched [11]. Due to the uncertainty of color combination acceptance by consumers, many designers give up on exploring new color schemes. In terms of research, only a few studies are related to the learning of traditional colors [12], and even fewer have focused on generating color schemes based on traditional colors [13]. Thus, understanding consumers’ acceptance of traditional color combinations remains a core concern due to its importance in the success of a product.

Prior research has suggested that visual product features, such as color, shape, and contrast, influence aesthetic judgment and the emotions of consumers [14], which affect their purchase decisions. Colors provide information about the surroundings and also transmit the symbolic meaning of a product [15]. For example, the colors red and orange are associated with energy and passion and are considered valuable for products aiming to evoke excitement and appetite. Similarly, the colors green and blue are associated with calmness, appropriate for brands aiming at product reliability and serenity. As such, colors have long been an integral part of marketing, but research is limited on color evaluation and the potential impact on product design [8]. A review reveals that the association between colors and psychology is worthy of exploration [16]. For example, Xu [1] noted that a study of the relationship between color and psychology can provide a solid foundation for the extraction and use of traditional color schemes that attract consumers. Hence, this study aims to investigate the user-color association according to the modern consumer’s emotional attachment and attitude toward color combinations which will be extracted from traditional colors.

Turkmenistan’s traditional decorative patterns are taken as the key research object of this article which represents five tribes and acts as a significant inheritance and symbol of the country. These patterns have a long history of formation and are used in a variety of national and traditional objects, such as the national flag, emblem, and carpets. In addition, traditional color as a cultural-based element has an impact on consumers’ psychological perception, thus, Turkmen’s traditional colors are an appropriate object for the purpose of this research.

The remaining parts of this paper include the following sections. We begin by providing relevant literature elaborating on the psychological influence of colors on user associations and color extraction. In the next section, we develop the research method, including the research sample, research objectives, color extraction method, and color-emotion-centered surveys. In the next section, we present the results of the data analysis and discuss color schemes on 3D models. Finally, we conclude with a discussion and limitations of the study.

## Literature review

### The psychological influence of colors on users’ emotions

Color offers dimensions like hue, saturation (chroma), and brightness (value), where it constitutes a visual effect caused by the stimulation of light. The studies researched on color’s influence on people, chiefly on children, found that visual impact has a tight link with individuals’ psychological feelings, like emotions and mood [17–20]. The reason that children prefer visual operations like using visual features of the object to process the information over verbal communication since they have not fully mastered their cognitive abilities at an early age [21]. Children are enclosed by a significant number of diverse colors from their daily environment, in particular toys. Furthermore, they can be under the influence of parental preference and color culture, as well as, gender-color stereotypes like “blue for boys, and pink for girls” [17, 22, 23]. Thus, children gain and develop their emotional knowledge through visual perception and interpretation of their environment. As a consequence, designers can use this opportunity to enhance the merit of selling by attracting consumers’ curiosity and stimulating customers’ willingness to purchase which might help to grow brand awareness.

The color associated with personal emotions and feelings can determine the expression of a specific color whether it is negative or positive according to the child’s preference. For instance, a child who had an undesirable incident with the color yellow will habitually treat it as a negative color, and vice versa. Nonetheless, the changes and variations in color notation and dimensions of single or multiple color combinations can affect how children will perceive a particular color [24]. Furthermore, another factor that influences children’s perception of a certain color and color-emotional attachment is gender difference. The gender analysis presented the differences between girls’ and boys’ rated colors positively or negatively, where solely pink and purple came to be negative but blue or red were positive for boys, and contrariwise for girls, whilst, there was no gender difference in the evaluation of other color shades (yellow, orange, white, blue, green, and black) among children [24]. In view of this concept, Jiang, Cheung [17], Jonauskaite, Dael [23], and Gyu “Phillip” Park [25] stated that gender differences in color preferences can be explained by color dimensions and the difference between the choice of these dimensions children make referring to their gender role. Firstly, referring to the views of this study, the total color preferences didn’t vary depending on the value (brightness) in any age range. In addition, as evidence, brightness or lightness with saturation leads to more positive emotions, which explains the direct relationship with an individual’s emotions [20, 25]. Secondly, girls preferred more saturated colors, but boys had a better preference for all levels (low, high, and achromatic colors) of the saturation scale, hence, explaining the theory of gender differences in psychological responses [17, 23]. Conversely, chromatic sense did not vary among adults’ preferences, but remained the difference between genders in favoring a particular color. Finally, in preference of hue dimension, children favorably select colors in the low blackness group [17, 23]. Considering the perceived results from numerous studies on an emotional connection to colors, users make a decision of favorite or most popular color in favor of blue, red, and pink.

Despite the stereotype of “blue for boys, pink for girls”, prior studies elucidate that the preference goes for blue no matter the gender or age contrast. Notably, female children of age 3 most probably will elect to choose pink or purple, nonetheless, after developing their cognitive abilities in a competitive environment with males, women try to eschew the pink color which shows their low status, since pink symbolizes the softness, tenderness, and emotional or immature nature. Yet, pink remains in the highly positive emotional stage of adults’ color perception, nevertheless, the “feminine” color has several factors that influence people’s lack of interest in it. Consequently, the influencing factors of color taste can also affect the color grouping, which may be altered throughout the individual’s development [18, 23, 26]. Along with gender differences, children may also face a transformation in their color taste due to cultural differences [21]. For some cultures, green represents “a new beginning”, similarly, it may symbolize “unity” or “peace” as well. Chinese culture values the red color for the meaning of “good luck”, on the contrary, the perception of this color can differ drastically from other cultures since it may elicit an unpleasant representation [21, 27].

Therefore, color and emotions can be explained by some of the particular associations between them, such as psychological reactions, cultural impact, gender differences, and two connected dissimilar concepts or senses like the synesthesia phenomenon. The synesthesia phenomenon occurs when an individual relates a sensory system or concept with a different sense and concept, that is, associating colors with senses [18]. An equally significant aspect of users’ emotional connection to colors is metonyms, considering that, metonymic reasoning explains a color connection with a related item, concept, emotion, or cultural value, to put it more simply, like an image that arises in the mind of individual’s direct associations with a color(“red-tomato”) or the situation in which an individual faces negative or positive emotion with precise color, that is, the color will attach to that emotion which he experienced, thus, generates indirect associations with color [28]. Consequently, children’s color emotional-physical associations become drastically and mutually related, in view of the fact that they gradually develop their visual, perceptual, psychological, and cognitive abilities from the influencing environment.

### Color extraction

Color is an integral part of a culture, interpreting the unique traits of each nation, eventually delivering certain emotions associated with cultural beliefs. Furthermore, color is an important design element that along with demonstrable physical features remains a strong arousal for individuals’ meaningful associations [29]. Besides, color can increase interest in the product, in particular, when it is associated with one’s culture [30], hence, it may generate substantial psychological responses. Considering the importance of the cultural aspect of color and its impact on the emotional association of customers, it is important for research and practice to investigate the extraction of culture-oriented colors and how they influence individual emotions. Thus, this study of color and culture association is intended to establish users’ emotional associations with colors and their preferences.

Previously, Xu [1] applied the PAD (Pleasure—Displeasure, Arousal—Nonarousal, and Dominance—Submissiveness), a three-dimensional model to define the relationship between different psychological perceptions of colors. To that end, MATLAB software was used to extract the color data from 20 representative Chinese traditional decorative patterns. Another study used a manually generated CYS color dataset using GAN multi-modal input, which contains categorized 1263 images. Subsequently, extraction occurred by clustering approach [31]. Furthermore, referring to a review of the literature related to the extraction methods of colors from images, studies have explored several methods, such as k-means and mean-shift clustering, fuzzy logic, a median filter of the image, the Chinese traditional Five Elements Chromatology, psychological testing using semantic differential analysis and others [32–34]. Besides, the extraction can be completed by available online platforms like Adobe Kuler, Image J, Adobe Color, and Adobe Hex.

The related studies mainly focused on extraction via psychological influence on individuals. Hence, according to the following literature, psychological-emotional or cultural attachment to colors remains a significant area in the art and design fields. In order to select an appropriate color for a certain product, designers must understand the factors that concern customers’ interests. In the case of the cultural aspect, the study should follow a color’s influence on Worship and Taboo, and consider elements like cultural characteristics and national color psychology. For instance, the Chinese traditional Five Elements Chromatology and its Tricolor Sub-aesthetic standard model apply the “Yin” and “Yan” philosophy, which contains morality and social qualities, along with cultural or national traits [30]. In addition, Xu [1] and Kuo and Lai [34] focused more on the psychological effects on human color perception to extract colors for further use, since they believe there is a tight link between an individual’s mind and color recognition. In light of the psychological approach, it is significant to note the primary explanatory characteristics of color-emotion associations, namely, color-emotion vocabulary or color-culture-emotion vocabulary to analyze an individual’s reaction. In order to generate color-emotion associations, Kobayashi [35] developed a color image scale via a three-dimensional space of psychological factors (soft-hard, warm-cool, and grayish-clear), where color combinations and emotional vocabulary have a mutual correspondence. Consequently, the comparable meaning of color combinations and emotional vocabulary is located at the nearest position on the color image scale, to elucidate the relation between colors and the user’s psychological expression, aside from, matching colors in order of psychological consequences and feedback [36]. Besides, the color integration may vary in color dimensions, and the determining color spaces in RGB, CIE Lab, and HSV, suggest that color-emotions vocabulary along with psychological feedback will change depending on a certain color tone. In view of apt color space for color detection, CIE Lab color space remains the most commonly used in scientific research, insofar as, the human visual system and comprehension of visual objects are similar to the perception through this color space. Nevertheless, the RGB color model may be appropriate, since it is nearly based on the tristimulus model of human color vision, thus, the distances approximately display perceived distances.

## Research methods

**Fig. 1:**
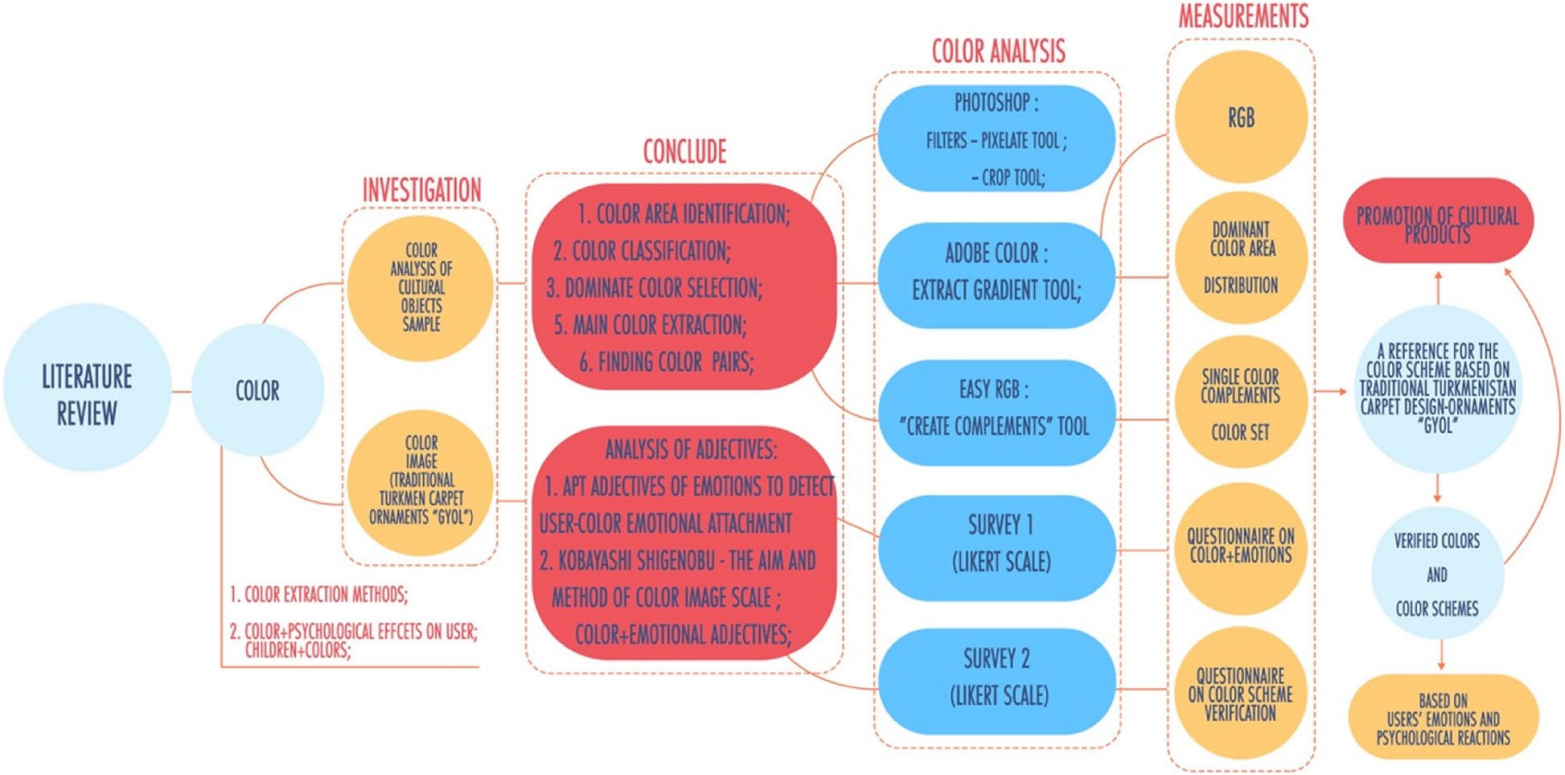
Research framework

We adopted a three-step approach to develop and test color schemes. First, colors were extracted from Turkmenistan’s national carpets. In the second step, the first survey was conducted to test the consumers’ affiliation with colors to develop color schemes accordingly. In the third step, a second survey was conducted to assess which color schemes are valued by the consumers to suggest designers. Figure 1 below reveals the approach used in this study starting from color extraction and investigation of the emotional association of individuals with extracted colors leading to the development of color schemes to discover consumer preference for color combinations of children’s product design.

### Sample and procedure

This study aims to establish a color scheme for sustainable children’s products considering user-emotion associations with product colors by using extracted colors from traditional geometric patterns of Turkmenistan’s national carpets. These patterns consist of traditional Turkmenistan colors and have a significant influence on people [37]. Therefore, we used these as samples for our research to prepare suitable color schemes for design by referring to the emotional attachment to established colors and providing samples of children’s products using prepared color schemes.

**Fig. 2:**
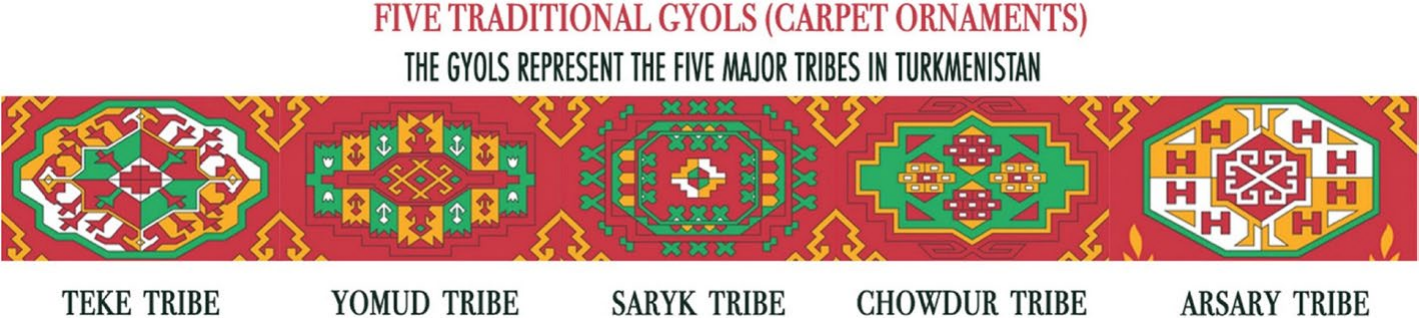
Turkmenistan carpet ornaments “Gyols”

Color extraction involves selecting five representative traditional geometric decorative patterns known as ‘Gyol’ from Turkmenistan’s national carpet. These patterns serve as examples to generate the color schemes. The surface design of Turkmenistan’s national carpet is covered with smooth and well-defined divisions of patterns of geometric origin, which are placed in staggered order. These “Gyols” are a repetitive eight-sided pattern representing the symbol of the Turkmen nation, as well as, attributed to the traditions and history of five tribes, namely, Teke, Yomud, Arsary, Chowdur, and Saryk-Salyr, as well as, the country’s nature, and related ancient totems, legends, and symbols. Moreover, the pattern’s design differs between color and shape by regional characteristics and attributes. Considering the color schemes of Turkmen carpets, red color dominates by ranging from dark dull red shades to vivid blood-red tones. Among others, as a rule, black, blue, white-beige ivory, and yellow tones can be found on carpets. Hence, five pictures of five carpet patterns were manually chosen from online websites by taking into consideration the characteristics of the five tribes and named using tribe names, respectively (Fig. 2).

### Extraction method

In this study, color extraction is accomplished through Photoshop, the color semantic tool Adobe Color, and Easy RGB color converter. The prepared images for extraction were edited in Photoshop. Adobe Color, previously, Adobe Kuler, generates color schemes from different techniques, such as, “Color Wheel” or solely color and color themes extraction tools, which are used to extract colors, generate color palettes, and create gradients. The default format is RGB, however, it can be transformed to CMYK, HSV, or LAB color space. “Easy RGB” web platform is employed to generate the desired color complements. Color detection is based on the use of a desktop spectrophotometer in the temperature control zone of a particular object. In light of the Munsell notation, generating and matching color data is implemented using a mathematical algorithm, whereas, the measures of all color samples can be determined in the color-matching service.

### Color scheme sample design

After the extraction of colors, this paper surveyed consumers in China (using the most representative adjectives about emotions) to analyze individuals’ psychological reactions toward certain colors, this helped the authors develop color schemes. In addition, the data analysis from a complete selection of 6 color hues and 124 color tones-shades for psychological testing using Likert scale analysis is carried out through the SPSS statistical tool.

### Verification of color scheme

After a month of the first survey, the second survey was conducted to validate the color schemes. In the second survey, 48 parents with preschool children (ages ranging from 2 to 5 years) were invited to report their responses about color schemes. The data analysis was performed using SPSS to verify the relevance of the color scheme design.

## Analysis and results

### Color extraction process

**Fig. 3:**
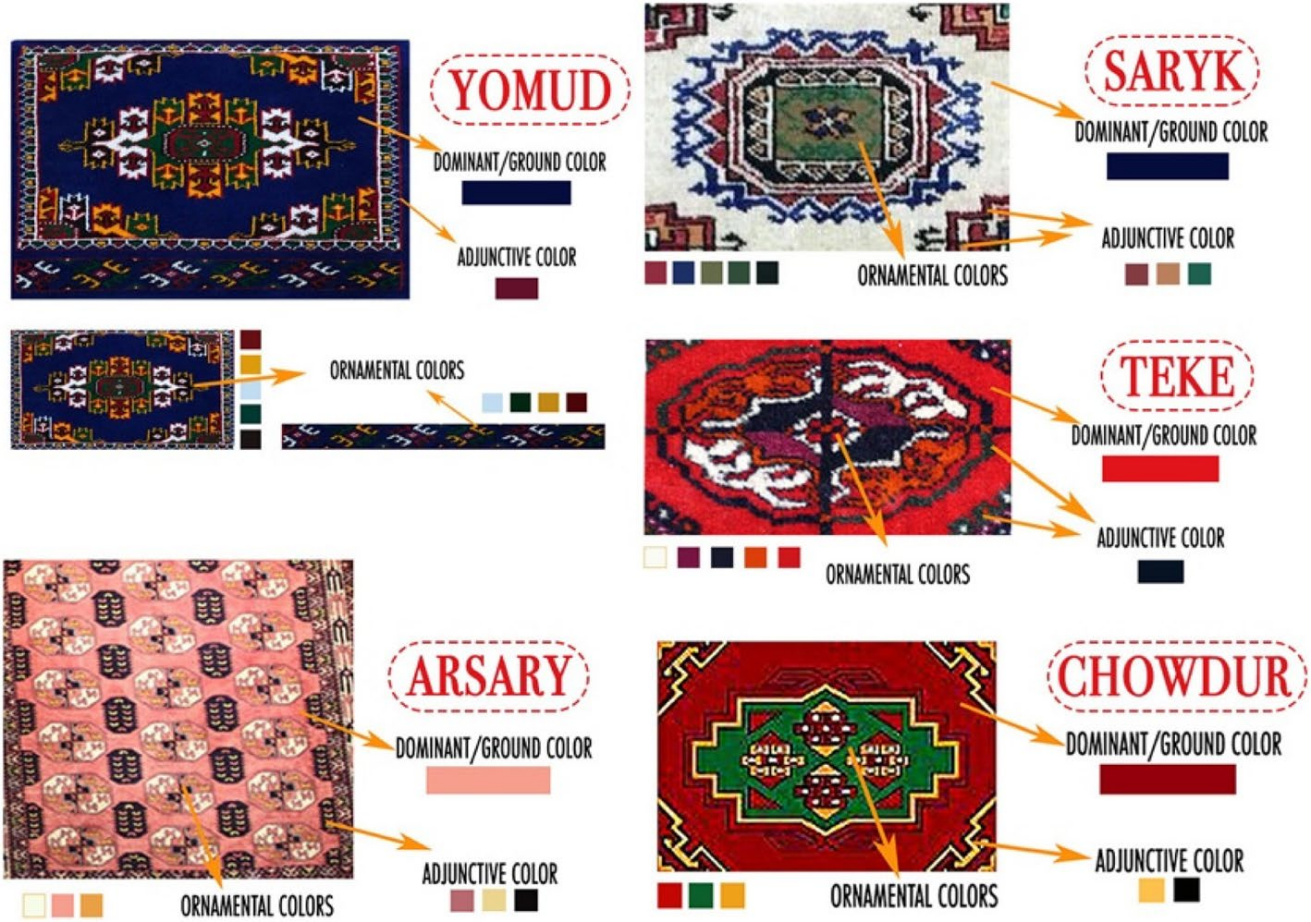
Images representing Turkmenistan’s 5 tribes

**Fig. 4:**
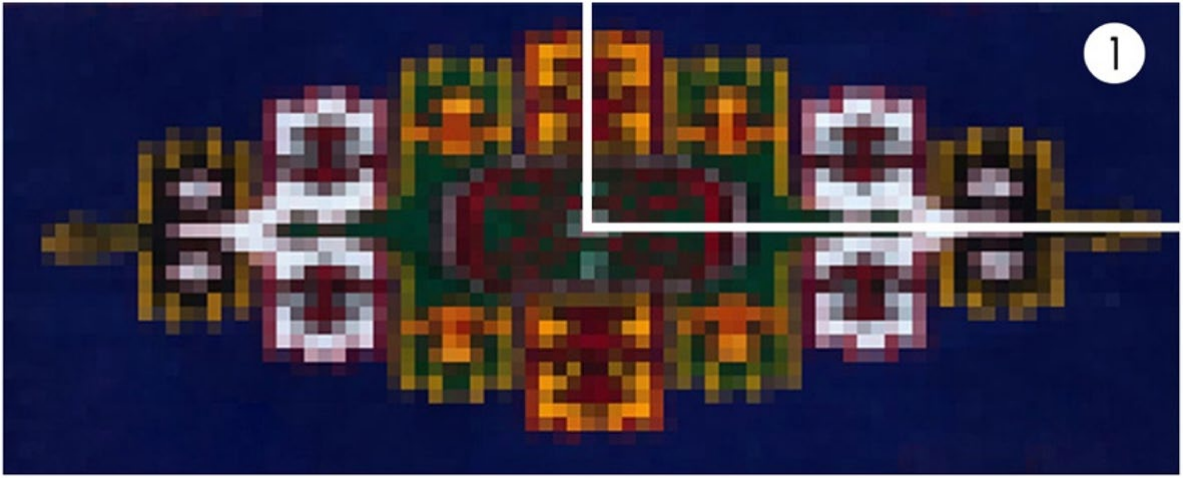
Division of one-color area for extraction

**Fig. 5:**
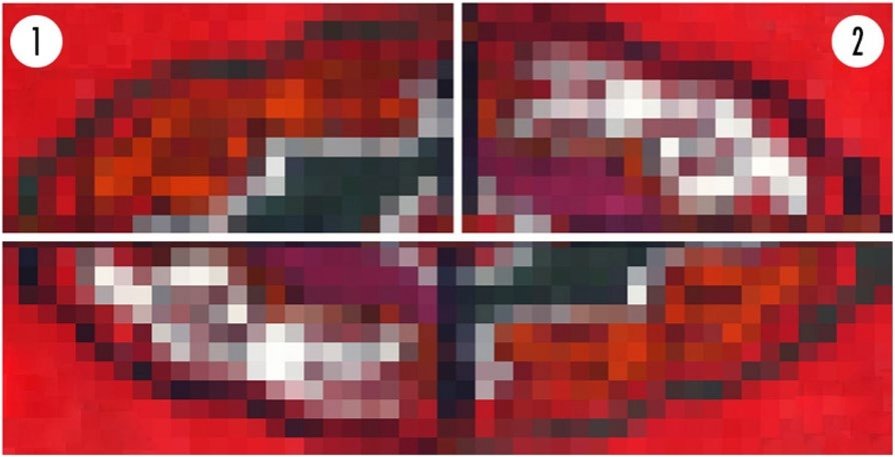
Division of two-color areas for extraction

With the aim of extracting the main colors of Turkmenistan’s traditional carpet patterns, this study applies five images of carpet ornaments relating to the five main tribes of the Turkmen nation, which are obtained from Internet resources presented in Fig. 3. Since color lines are adjacent to the surrounding colors, a procedure was undertaken to separate color shades by mosaic pixelation of a specific department in Photoshop to highlight/accent the dominant color. In the process of mosaic pixelation in Photoshop, a pixel size range of 15–20 was chosen. Due to the fact that these patterns are geometric figures, it was decided to divide them into 4 parts and use one part in case the colors in the pattern did not change presented in Fig. 4. However, if the pattern was generated from two different color combinations, it was divided into two principal parts to derive the color presented in Fig. 5. Consequently, 2 groups were established including two parts of the geometric pattern (Teke, Arsary), as well as, 3 groups of one part (Yomud, Chowdur, Saryk).

To extract colors, the “Extract Gradient” function of the Adobe Color online generator is implemented, providing an RGB color value. Using this tool, 10 stops were made on a specific color from the main color, sub-color, and accent color, which was edited manually and set on the color by groups. For the content of the main color in a particular part of the ornament, 50% of the total surface was delivered; sub-color, from 10 to 50%; and accent color, less than 10%. Adobe Color supports the use of up to 15 stops for extraction; however, a decision was made to utilize only 10 stops to achieve enhanced clarity and precision. The color groups were assembled to analyze the characteristics and location of the colors, such as primary colors (red, yellow, blue), secondary colors (green, cyan, purple, orange), and composite colors (brown). Moreover, white and black-dark selections on the image were named light shades and dark shades of a particular color (blue, yellow, red, purple, brown). After the color classification, 11 main colors (warm and cool colors) and 220 color shades and tones were determined. Extracted colors were challenging to differentiate from each other, thereby, it was decided to select colors through the method of removing similar and redundant colors that cannot be easily distinguished. As a result, 110 color shades were obtained, precisely half of the initial number. The guiding principle for selecting the highlighted colors was their applicability to children’s products, a choice informed by a thorough review of color usage in existing products for children, as discerned from online references. This deliberate and considered approach ensured that the final selection of colors was not only visually coherent but also resonant with the intended audience and purpose.

After this procedure, the “Easy RGB” color data search engine is used on the basic mathematical equation and calculations, for the sake of preparing color swatches using one specific entered color data. Accordingly, contact color data is entered using the RGB space, which is automatically set by the illuminator and the ambient observer (D65/2°). All automatically generated secondary colors and the main color will be displayed in the RGB value. For a more accurate process to create a presentable color palette in the use of children’s product design, color compositions of 1320 colors were reduced by removing colors that are very close to similar. First, complete color groups were transferred to Keyshot to compare colors by applying colors to 3D objects and realistic visualization. Next, on the basis of the resulting rendered image, resembling colors were removed via processing similar colors on the color gamut, along with entering a numerical value in the text fields R, G, and B. Thus, 457 less or more saturated color shades and tones were selected. In addition, the color scheme generation process was carried out. To this end, the following rules had to be taken into account: (1) removing identical and undifferentiated colors; 2) apt color selection for use in children’s products. Consequently, of the 457 colors, only 140 representative colors were left, divided into groups under the name of each tribe, as well as subgroups by color (red-pink, green-blue-blue, purple, yellow-orange-.

### Surveys sample overview

The first survey consisted of 104 parents including 51 men and 53 women who have children of preschool children from China. The survey was translated into Chinese language. All 104 participants completed the survey on color and color scheme preferences. The majority of participants were between the ages of 18 to 25. For the second survey, 48 parents of preschool children, including 22 males and 26 females aged 25–35 were invited.

#### Survey 1

In order to prepare a color scheme for use in children’s product design, the first survey was conducted on the emotional-psychological connection between color and user and how color influences a user’s feelings. The survey items were adapted from Ackerman [38]. The investigation of emotional characteristics of values and hues of color has been made. The prepared and already chosen color groups were performed to study the emotions provoked by users meeting with a certain color. Before conducting the questionnaire, this study researched which color hues most appeal to the consumers of children’s items. Subsequently, it was selected 124 colors of 6 color groups – red, yellow, orange, purple, green, and blue. First, it was asked to complete the questions related to the emotional expression of the user towards a specific color. Using the Likert scale, it was found that the favorite colors of preschool children were red and yellow, differing in gender. Besides, among the given 6 color choices, boys favored red (28.8%), orange (23.1%), and green (19.2%), whereas, girls preferred yellow (26.9%), red (25%), and orange (19.2%), see Table 1.

**Table 1:** Q6 Children’s color preference

Gender	Color	Frequency	Percent	Valid Percent	Cumulative Percent
Female	Red	13	25.0	25.0	25.0
	Blue	5	9.6	9.6	34.6
	Green	6	11.5	11.5	46.2
	Yellow	14	26.9	26.9	73.1
	Orange	10	19.2	19.2	92.3
	Purple	4	7.7	7.7	100.0
	Total	52	100.0	100.0	
Male	Red	15	28.8	28.8	28.8
	Blue	3	5.8	5.8	34.6
	Green	10	19.2	19.2	53.8
	Yellow	5	9.6	9.6	63.5
	Orange	12	23.1	23.1	86.5
	Purple	7	13.5	13.5	100.0
	Total	52	100.0	100.0	

The survey established that red and orange were preferable to other choices in both genders, with boys electing red while girls chose yellow as their favorite color. However, the most unpopular colors were blue (boys 5.8%) and purple (girls 7.7%). Children did not find the colors blue and purple attractive and interesting. Nevertheless, it was asked for the parental preference of a color value of a certain color, such as color saturation, lightness, and darkness. Since parents tend to purchase the product for their children, hence, this point remains remarkable to success in the production business [17]. Consequently, the parental choice was stopped in 59 color codes as shown in Table 2.

**Table 2:**
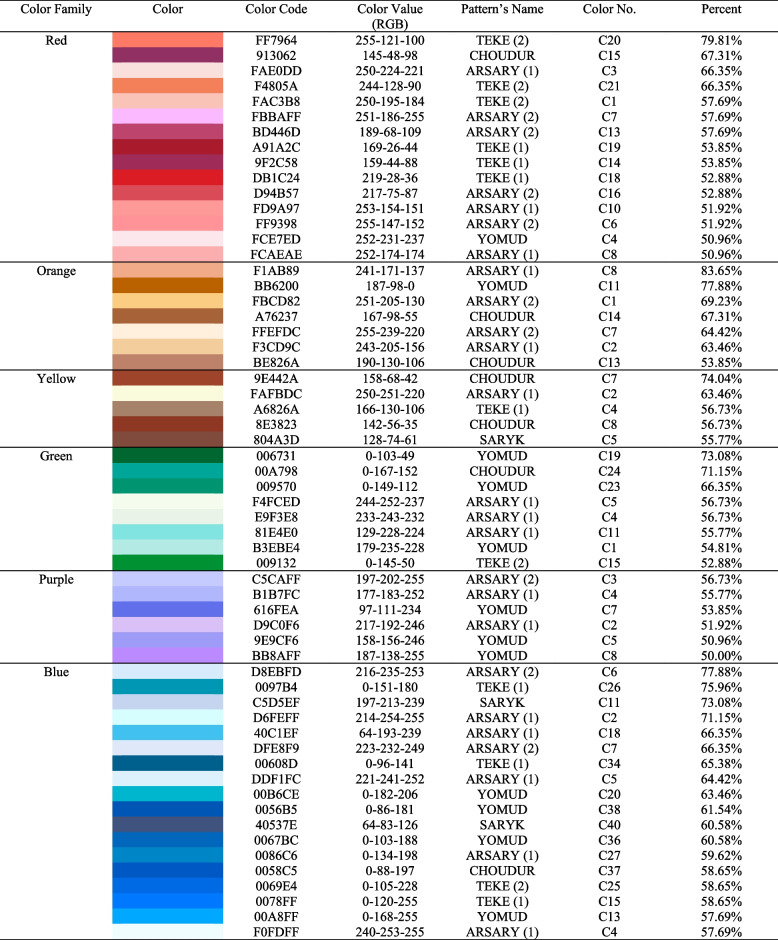
Q19-24 Parental color selection for the design of color schemes of children's products

**Fig. 6:**
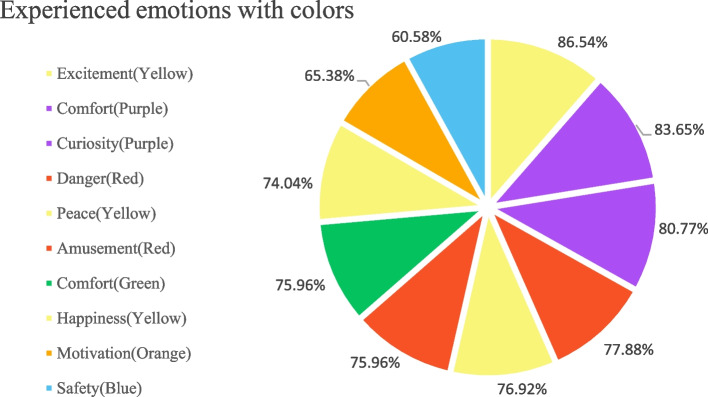
Q13-18 Color-emotional associations

Next, to examine the color association with children’s psychological perception, it was required to answer the question in the following order: (1) Positive; (2) Negative; (3) Neutral; (4) Mixed, and (5) Other (space for participant’s opinion). As a result, children had the most positive results with red (64.42%) color than orange (30.8%), yellow (26%), green (17.3%), purple (14.4%), and blue (9.6%), sequentially. Regardless of the literature, this study detected that the blue color had the least preference (boys 5.8%, girls 9.6%) and the least positive emotions (9.6%) in children since they perceived this color as neutral (51.9%). Moreover, green (none 43.3%), yellow (35.6%), and purple (30.8%) have not delivered any impact on children’s color-emotion associations. Thus, in order to understand how a certain color will impact a user’s emotions, it was decided to conduct a question on children’s color experience with proper adjectives. Through the literature review, after the filtration process, 18 adjectives that are suitable for testing psychological association with color were generated. The results showed that the most popular adjectives were in such order as excitement (yellow 86.54%), comfort (purple 83.65%; green 75.96%), curiosity (purple 80.77%), danger (red 77.88%), peace (yellow 76.92%), amusement (red 75.96%), happiness (yellow 74.04%), motivation (orange 65.38%), and safety (blue 60.58%) (Fig. 6). Consequently, the obtained results of emotional experience and user associations on the basis of the estimated factors were taken to further selection and color scheme verification.

#### Survey 2

**Fig. 7:**
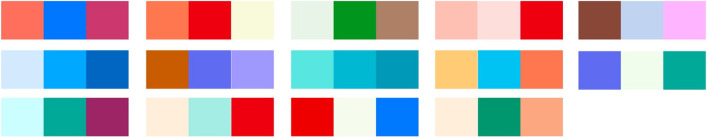
14 color schemes - tricolor combinations

**Fig. 8:**
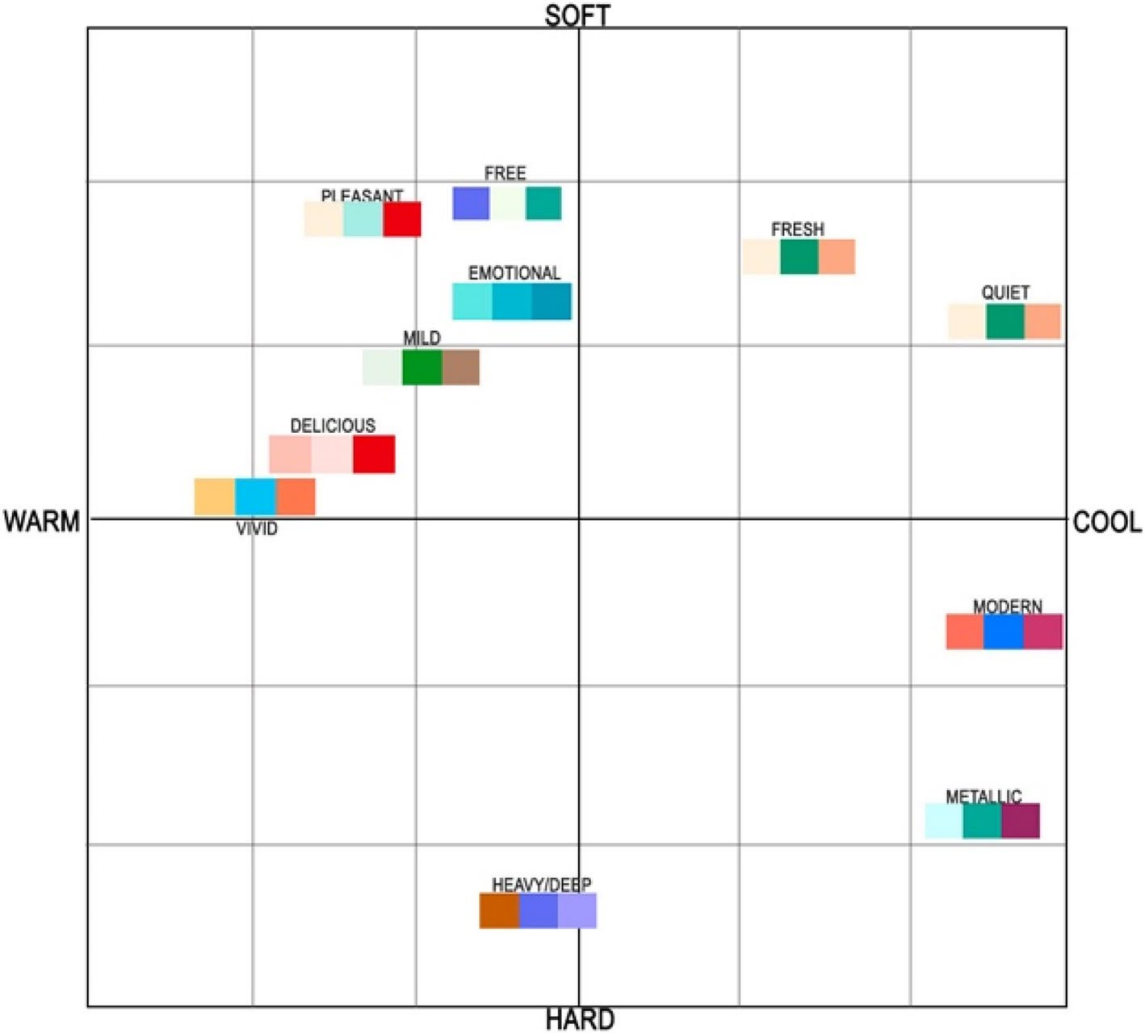
Verified color schemes on Kobayashi’s color scale

After obtaining a particular number of colors for children’s products according to the findings of survey 1, colors varying in dimensions such as lightness-blackness, and brightness-saturation were transferred for the enduring color combination process. Next, survey 2 was conducted on color scheme generation using precise adjectives based on 180 emotional words from the Kobayashi color system [35, 36]. developed an image scale to produce color schemes by applying emotional words. He conducted a survey process to establish three color combinations on the soft/hard and warm/cool color scales. Thus, in his work, he developed 900 pairs of three-color combinations that have equal meaning with the established 180 emotional words on a two-dimensional color image scale. In light of color scheme verification, it was applied the evaluated emotional qualities of various color hues and tones to the tricolor combinations in the second questionnaire. For this experiment, the paper selected 74 emotional words from Kobayashi’s emotional word dataset, representing users’ emotional attachment to the prepared three-color combinations. Considering the first survey results, 14 color combinations – color schemes are assembled to research the emotional feedback of a user with a certain color set in order to find the accurate/veracious scheme for children’s product design. The following color combinations are shown in Fig. 7. Since parents are general clients, they contributed to the color scheme preferences in the second survey. A total of 22 questions were included, with 14 tricolor combinations to generate well-defined and respective emotional words in order to understand the user’s psychological preference regarding the color set for the benefit of product promotion. To this end, two following questions were asked: (1) Please rate the following color scheme according to your preference; (2) Please choose proper adjectives for the following color set. Initially, participants expressed their color preferences, providing data for color set verification. Subsequently, based on individual responses, relevant emotional descriptors for the color data were generated for use in the color combination image scale. In total, this experiment established 11 adjectives evaluated by their relevance to the current study and chosen color schemes, of which the results are shown in Table 3. Kobayashi’s color combination image scale consists of soft/hard and warm/cool two-dimensional spaces, where color sets relevant to each other are grouped into 13 categories [35]. Subsequently, these two questions supported the discovery of the applicability of prepared color schemes in these two-dimensional spaces by categories. As a result, the location of the tricolor combination on the specific areas of the color combination image scale was determined shown in Fig. 8.

**Table 3:**
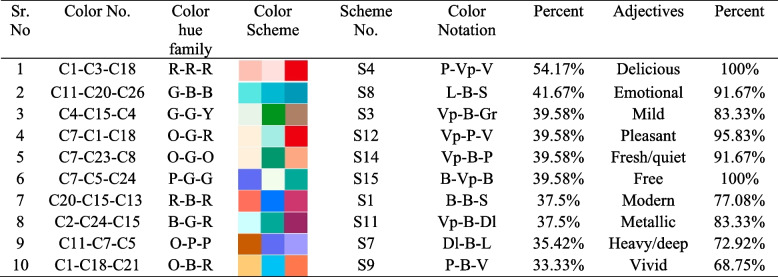
Color scheme and suitable adjectives verification

R-red; G-green; B-blue; Y-yellow; P-purple; O-orange; P-pale; Vp-very pale; V-vivid; B-bright; Dl-dull; S-strong; L-light

### Verified color scheme samples

During the survey process, data on the color scheme and colors were developed and established. Using color matching techniques such as complementary colors and similar color combinations, this work combines primary, secondary, and tertiary colors into three-color combinations to generate color schemes. The color and color set formation process was achieved by generating the Likert scale questionnaire using extracted colors from Turkmenistan’s national carpet ornaments with valid emotional words. Thus, the results revealed that pale pink, blue, green, orange; very pale pink, green, yellow, blue; light grey-green, orange, green, purple, blue; deep red, green, blue; strong blue, green; grayish yellow, orange; dull yellow, orange, green; bright purple, red, blue, green; and vivid red, blue color hues had the highest preference and attractiveness among the users. Moreover, users preferred 11 color schemes out of 15 with the range of 54.17% − 33.33%, where 54.17% was the highest mark and 12.50% was the lowest.

In addition, reference images from online platforms, specifically Pinterest, were utilized to develop color schemes. To curate a relevant database, keywords such as “Children’s furniture,” “Toys,” and “Kids’ bicycles” were employed. Another avenue of research was the analysis of existing literature. A study by Jiang, Cheung [17] revealed distinct color preferences: girls favored pink and purple, boys preferred red, and both genders showed a preference for blue. The study highlighted a general preference for red and blue over green and yellow, especially in low-blackness colors. Furthermore, it underscored gender differences in color preferences, with males showing a greater inclination towards high chromatics, low chromatics, and achromatic colors compared to females.

These findings were instrumental in shaping the color schemes for children’s products, taking into account the nuances of gender-specific preferences, the psychological impact of color brightness and chromatics, and the cultural and emotional associations with certain colors. This comprehensive approach ensured that the selected color schemes were not only aesthetically pleasing but also aligned with the preferences and psychological responses of the target audience.

## Implications and conclusion

### Application of the color scheme prepared by using the representative colors of Turkmenistan in the design of children’s products

#### Implementing color scheme in toy production

**Fig. 9:**
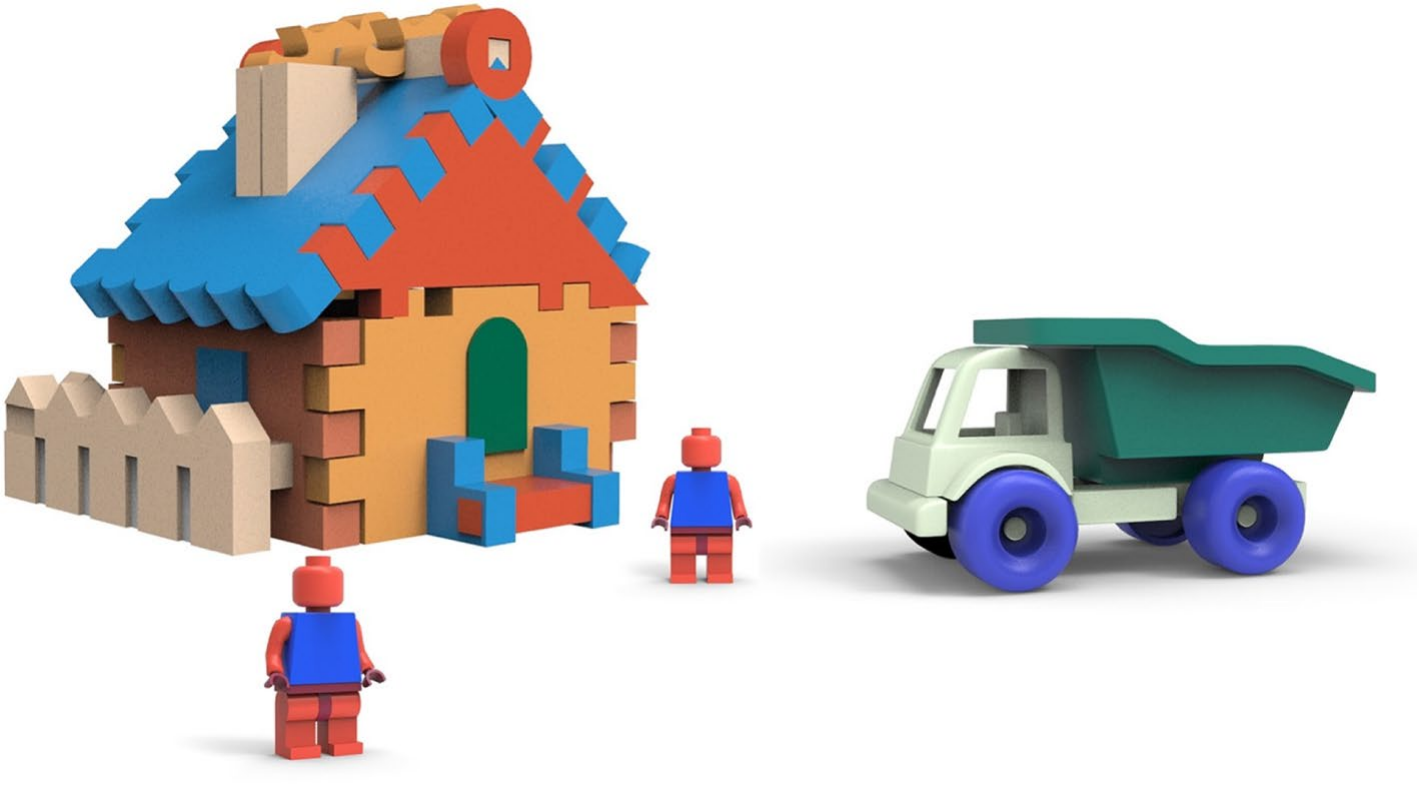
Implementing color schemes in toy design

In view of color choice expediency, this paper employed designed and deliberate color schemes on 3D models which are prepared by using Keyshot and Rhinoceros software. Color schemes were represented on the basis of color suitability and a particular model. Furthermore, several schemes were combined in one model to emphasize the aesthetic appearance of the toy in greater detail. In this process, S1(a man), a combination of S9 and S14 (a toy house), as well as S15 (a truck) color schemes were used in Fig. 9.

#### Implementing color scheme in furniture design

**Fig. 10:**
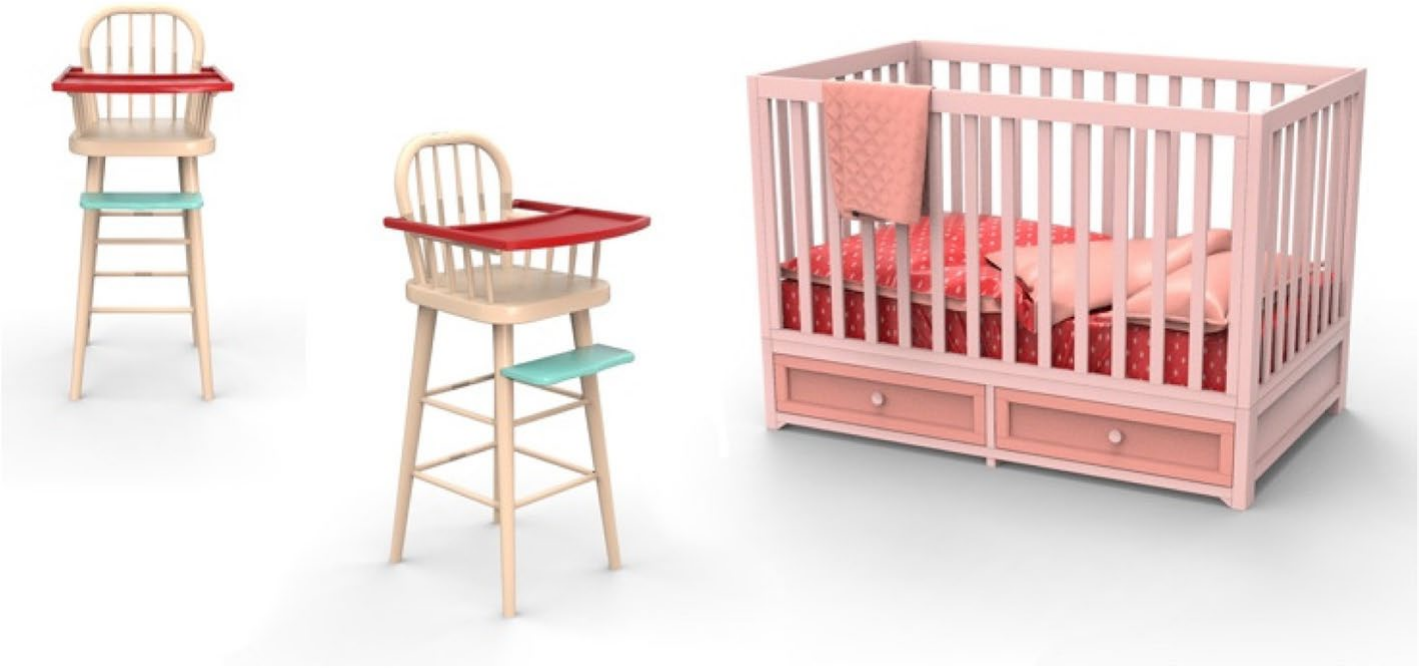
Color schemes used in furniture design

Based on the literature review, for furniture color preference children prefer low blackness, achromatic, high saturated, and unmuted or muted colors [17]. Furthermore, the favored color hues were red, yellow, and orange, respectively, but disliked blue hues. According to modern consumer preferences, light shades such as light yellow, light blue, and light red hues were established as favored colors for any type of furniture. However, it was discovered that children’s color preferences may change depending on their age. Thereby, the color saturation decreases, and color hue selection increases. Besides, the color appropriateness of items may depend on individuals’ life aspects like cultural context, emotions, color associations, etc. Thus, based on this study, the following color schemes were used: S12 (a dining chair), and S4 (a bed), which are shown in Fig. 10.

#### Implementing color scheme in product design

**Fig. 11:**
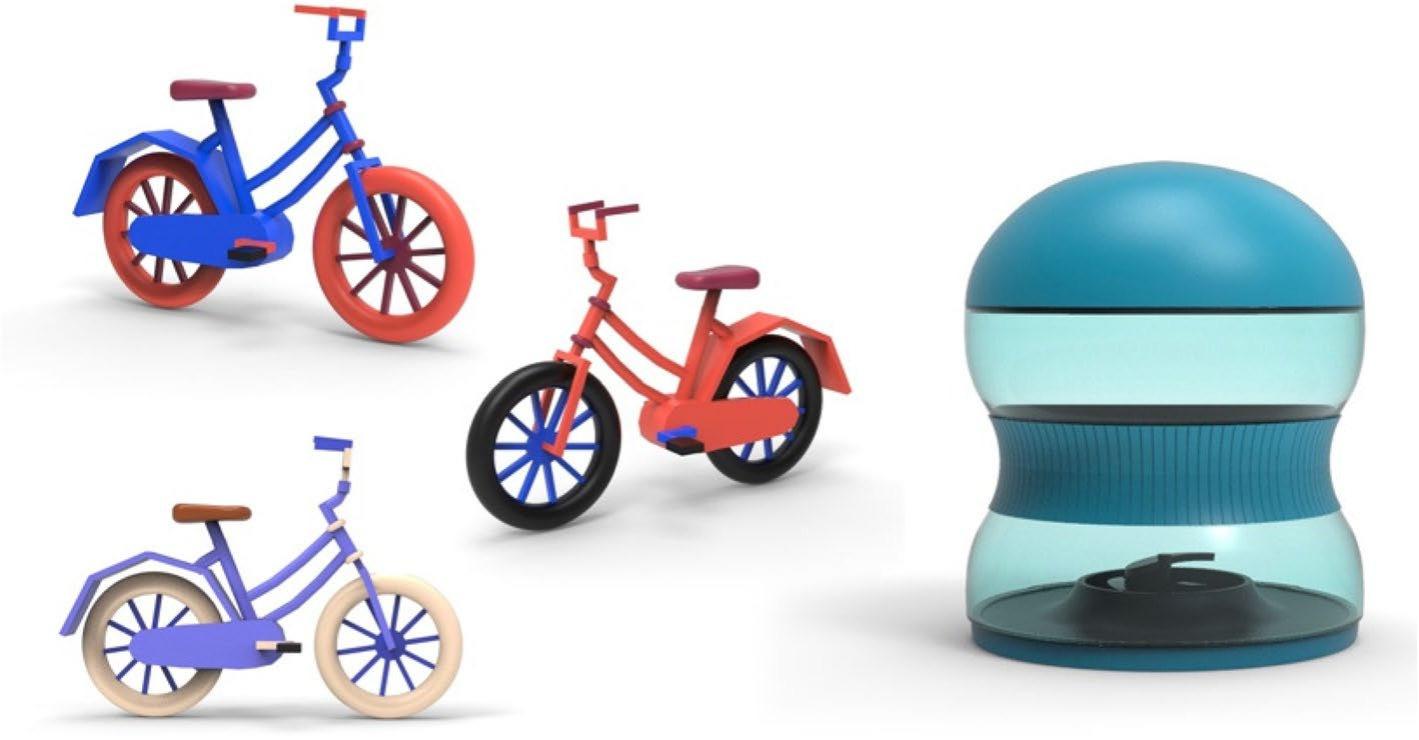
Color schemes used in product design

The color scheme was chosen for use in the design of children’s products, and distinctions were also considered, including the aesthetic appearance of products and popular modern colors in the field of design. Thus, 3D models were prepared to show the selected color schemes using the following color schemes: S1 (a bicycle), S1 and a color black (a bicycle), a combination of S7 and C7 (a bicycle), and S8 (a baby bottle), which are shown in Fig. 11.

### Theoretical implications

Traditional decorative patterns remain precious and unique to the cultural identity and heritage of nations and their people. Colors take a dominant position in these patterns due to their inherent cultural and symbolic significance, profoundly influencing the psychological perceptions of individuals. The use of these traditional colors in product design can influence aesthetic appeal and consumer valuation of products, anchoring their traditions and culture.

In this era, marked by rapid advancement in technology and globalization, designers and manufacturers often face the challenge of offering innovative products that not only attract but also retain customers. It is well-established that color plays a pivotal role in consumer purchase decisions. This prompted designers to pay close attention to color schemes in product design. Research has suggested that traditional color schemes are vital for designers to offer products aiming at resonating with consumer’s expectations [33]. However, the integration of traditional colors into product design does not always yield the anticipated benefits, as consumer appreciation for these colors can vary. As such, designers and manufacturers are increasingly focusing on refining the use of traditional color schemes in products. Indeed, if color schemes are properly used, can fulfill consumer expectations, fostering a sense of national identity, cultural connection, and shared values. In line with prior research, our study contributes to the literature that underscores the significant impact traditional color schemes can have on consumer purchasing behavior.

This study took Turkmenistan’s traditional decorative patterns as a research sample. These traditional decorative patterns are chosen for their profound symbolic significance and historical importance to the country and its people. There are enduring connections between Turkmenistan and China, providing a rich context for our research. These relationships are strengthened over centuries through the Silk Road, with Mary, formerly Merv, serving as a pivotal trading city for Chinese merchants on their route to Europe. The shared national color of red between Turkmenistan and China further underscores the cultural and historical ties between these nations, making Turkmenistan’s traditional patterns a particularly insightful sample for examining color preferences among Chinese consumers. Embodying centuries of cultural heritage, these traditional patterns influence the psychological perceptions of the people, making a valuable sample for our study. The findings of this study suggest that innovative color schemes derived from these traditional patterns when used properly in product design, can enable organizations to meet and exceed customer expectations. As a result, color schemes extracted from traditional decorative patterns enhance product value and contribute to the overall success of the business.

In line with prior research, for example, Xu [1] explored the color extraction from Chinese traditional patterns, Martinez, Rando [3] studied the impact of packaging color on consumer purchase intentions, and Elliot and Maier [16] studied color psychology and human behavior, our study further confirms the critical role of traditional color schemes in shaping consumer purchase decisions. Our study findings reveal that by embracing traditional patterns to extract color schemes, companies can forge a stronger connection with their target audience, reflecting and honoring their national identity, culture, and values, thus offering products that are not only aesthetically pleasing but also culturally meaningful.

### Research limitations and future directions

This study has some limitations which create opportunities for future research. First, regardless of the precise L*, a*, and b* color data, this study employs the sRGB color value since the color generation was produced using online color generators. These programs cannot perfectly represent colors due to some obstacles such as differences in hardware characteristics (screen, monitor), environment, printed coating, materials, and settings on the device. Due to the fact that the color gamut on the computer is limited, the colors that are applied to the product may vary due to the above reasons. However, colors were displayed with good accuracy. For future work, designers can use the listed methods in order to obtain an accurate color extraction method.

Second, another key fact to remember, the indicator of color preference may vary altering on demographics, cultural preferences, and taboos. Asian societies, such as China have unique cultural characteristics that differ from Western countries’ color preferences. Therefore, future scholars can replicate these findings in other cultural contexts.

Third, in order to generate color combinations, 25 colors out of 59 were applied, relating to the following aspects: highly ranked colors according to the choice of users, the choice of color by the designer, application of “six colors” in tricolor combinations, and modern trends in the color schemes of children’s products. In addition, these color combinations were prepared for particular 3d models, thus, model characteristics were also taken into consideration during the color scheme establishment. Therefore, for the current study, only 14 color schemes were established using the extracted colors verified by the user’s color preferences, resulting in the selection of 11 color schemes. However, the 59 extracted colors can be used and combined into different color associations.

### Conclusion

Color not only conveys a product’s features and characteristics while remaining attractive to customers but can also trigger a positive or negative psychological perception of a particular product [8]. Traditional colors can be treated as an inheritance of culture, which is considered the symbolic representative of nations [1]. Therefore, cultural design can enhance the aesthetics of a product, enriching its meaning to establish great business opportunities that will develop brand awareness. To this end, designers employ traditional color schemes in design, however, many of them fail to attain popularity, since the colors they use are outdated and beyond the modern tastes of the users. In accordance with this problem, this article took Turkmenistan’s national and traditional carpet decorative ornaments – “Gyol” as the research samples. Firstly, the color extraction method was developed using traditional color hues and color data from carpet patterns. In order to prepare main color compositions and color scheme samples, this study conducted a process to explore the relationship between traditional colors and user color emotional response based on psychological preference. This study identified color preferences among Chinese consumers. Contrary to traditional stereotypes associating blue with boys and pink with girls, our findings reveal that boys showed a preference for red over blue, while girls favored yellow over red. However, considering the collective response of males and females, empirical findings suggest that the red color is the most favorable among both genders. According to the results of the first survey, a second survey was generated, which used 14 prepared color schemes to discover users’ preferences for given color schemes. Thus, this study provides guidelines to the designers on which color should be used in color combinations in order to capture the attention of the consumers. Furthermore, the results revealed 10 color schemes with 11 emotional words from the Kobayashi color image scale and 60 color codes for use in children’s product design.

### Supplementary Information

**Supplementary Material 1.**

## Data Availability

The datasets generated during and/or analyzed during the current study are available from the corresponding author upon reasonable request.

## Declarations

Prior to conducting the surveys, informed consent was obtained from all study participants and/or their legal guardians to ensure their willingness to participate in this research.

## References

[1] Xu B (2021). The inheritance and creative design of traditional color scheme based on modern consumer’s psychological perception: taking Chinese traditional decorative pattern’s color collocation as an example. Color Res Appl.

[2] Chai C (2015). The relative effects of different dimensions of traditional cultural elements on customer product satisfaction. Int J Ind Ergon.

[3] Martinez LM (2021). True colors: consumers’ packaging choices depend on the color of retail environment. J Retail Consumer Serv.

[4] Bagchi R, Cheema A (2013). The effect of red background color on willingness-to-pay: the moderating role of selling mechanism. J Consum Res.

[5] Madden TJ, Hewett K, Roth MS (2000). Managing images in different cultures: a cross-national study of color meanings and preferences. J Int Mark.

[6] Kpossa MR, Lick E (2020). Visual merchandising of pastries in foodscapes: the influence of plate colours on consumers’ flavour expectations and perceptions. J Retail Consumer Serv.

[7] Singh S (2006). Impact of color on marketing. Manag Decis.

[8] Kim D, Hyun H, Park J (2020). The effect of interior color on customers’ aesthetic perception, emotion, and behavior in the luxury service. J Retail Consumer Serv.

[9] Boyatzis CJ, Varghese R (1994). Children’s emotional associations with colors. J Genet Psychol.

[10] van Esch P, Heller J, Northey G (2019). The effects of inner packaging color on the desirability of food. J Retail Consumer Serv.

[11] Xu J (2022). A VR experimental study on the influence of Chinese hotel interior color design on customers’ emotional experience. Buildings.

[12] Choi I-R, Bang H-K, Kim Y-J (2008). A study on traditional colors. Res J Costume Cult.

[13] Zlatev Z (2019). Analysis on colors of folk costume and their application in contemporary textile design. Annals Univ Oradea Fascicle Textiles Leatherwork.

[14] Schnurr B, Stokburger-Sauer NE (2016). The effect of stylistic product information on consumers’ aesthetic responses. Psychol Mark.

[15] Krishna A, Cian L, Aydınoğlu NZ (2017). Sensory aspects of package design. J Retail.

[16] Elliot AJ, Maier MA (2014). Color psychology: effects of perceiving color on psychological functioning in humans. Ann Rev Psychol.

[17] Jiang L (2020). The impact of color preference on adolescent children’s choice of furniture. Color Res Application.

[18] Terwogt MM, Hoeksma JB (1995). Colors and emotions: preferences and combinations. J Gen Psychol.

[19] Aboubaker Ettis S (2017). Examining the relationships between online store atmospheric color, flow experience and consumer behavior. J Retail Consumer Serv.

[20] Zentner MR (2001). Preferences for colours and colour–emotion combinations in early childhood. Dev Sci.

[21] Zhang D (2019). Color versus form: which matters more in children’s preferences of package design?. J Int Consumer Mark.

[22] Gao Y (2021). Design of children’s product packaging preference based on big data machine learning. Wirel Commun Mob Comput.

[23] Jonauskaite D (2019). Pink for girls, red for boys, and blue for both genders: colour preferences in children and adults. Sex Roles.

[24] Pope DJ, Butler H, Qualter P (2012). Emotionalunderstanding and color-emotion associations in children aged 7–8 years. Child Dev Res.

[25] Gyu P, Park J (2014). Correlations between color attributes and children’s color preferences. Color Res Appl.

[26] Fateminia M, Ghotbabadi TD, Azad KM (2020). Perceptions of the taste of colors in children and adults. Color Res Appl.

[27] Hurlbert AC, Ling Y (2007). Biological components of sex differences in color preference. Curr Biol.

[28] Gollety M, Guichard N (2011). The dilemma of flavor and color in the choice of packaging by children. Young Consumers.

[29] Sherin A (2012). Design elements, Color fundamentals: A graphic style manual for understanding how color affects design.

[30] Zhou YX, Yang LL (2010). An attempt of using the national color culture on product design. Appl Mech Mater.

[31] Li Y, Zhuo J, Fan L, Wang HJ. "Culture-inspired Multi-modal Color Palette Generation and Colorization: A Chinese Youth Subculture Case". 2021 IEEE 4th International Conference on Multimedia Information Processing and Retrieval (MIPR), Tokyo, Japan. 2021. pp. 382-385. 10.1109/MIPR51284.2021.00071.

[32] Gunduz AB (2021). A better way of extracting dominant colors using salient objects with semantic segmentation. Eng Appl Artif Intell.

[33] Xing L (2018). Intelligent recognition of dominant colors for Chinese traditional costumes based on a mean shift clustering method. J Text Inst.

[34] Kuo LW, Lai CC (2019). Examining the color, size, and packaging design of wireless-mouse products. Color Res Appl.

[35] Kobayashi S (2009). Color image scale.

[36] Kobayashi S (1981). The aim and method of the color image scale. Color Res Appl.

[37] Tehrani J, Collard M (2002). Investigating cultural evolution through biological phylogenetic analyses of Turkmen textiles. J Anthropol Archaeol.

[38] Ackerman A. Color Psychology Questionnaire Results 2017 Available from: https://storybehindthecloth.com/color-psychology-overview-and-questionnaire-results/. Cited 2021 February 2021

