# Correction: Harmonizing culture and consumer psychology: optimizing color schemes for children’s product design inspired by traditional ornaments

**DOI:** 10.1186/s40359-024-01711-y

**Published:** 2024-04-18

**Authors:** Linglin Liang, Nazik Hangeldiyeva

**Affiliations:** School of Art and Design, Zhejiang Sci-Tech University, Hangzhou, China

**BMC Psychology (2024) 12:161**

10.1186/s40359-024-01644-6

Following publication of the original article, the authors flagged that the name of the first author, Linglin Liang, had been incorrectly provided as Liang Ling Lin. The name has now been corrected in the article. The authors thank you for reading this erratum and apologize for any inconvenience caused.

