# Supplementary material for: Harmonizing culture and consumer psychology: optimizing color schemes for children’s product design inspired by traditional ornaments
Source: BMC Psychol. 2024 Mar 18;12:161. doi: 10.1186/s40359-024-01644-6 (PMC10949696; doi:10.1186/s40359-024-01644-6)
Supplement: Supplementary file 1 — Supplementary Material 1. [file 40359_2024_1644_MOESM1_ESM.pdf]

Survey 1

1. What is your gender?
    - Male
    - Female
2. What is your age?
    - 18~25
    - 25~35
    - 35~45
    - Above 45
3. Do you have children?
    - Yes
    - No
    - If you have no children, please stop the questionnaire and leave the page
4. What is the age of your child?
    - Under 1 year old
    - 2~5
    - 5~13
    - Above 13
5. What is your child's sex?
    - Male
    - Female
6. Which color is your child's favorite?
    - Red
    - Orange
    - Yellow
    - Green
    - Blue
    - Purple
7. What are your child's feelings associated with the color red?
    - Positive
    - Negative
    - Neutral
    - Mixed
8. What are your child's feelings associated with the color orange?
    - Positive
    - Negative
    - Neutral
    - Mixed
9. What are your child's feelings associated with the color yellow?
    - Positive
    - Negative
    - Neutral
    - Mixed
10. What are your child's feelings associated with the color green?
    - Positive
    - Negative
    - Neutral
    - Mixed
11. What are your child's feelings associated with the color blue?
    - Positive
    - Negative
    - Neutral
    - Mixed
12. What are your child's feelings associated with the color purple?
    - Positive
    - Negative
    - Neutral
    - Mixed
13. Select all emotions your child has experienced with the sight, or thought, of the color red:
    - Happiness
    - Sadness
    - Fear
    - Anger
    - Boredom
    - Excitement
    - Amusement
    - Peace
    - Love
    - Hate
    - Playfulness
    - Safety
    - Danger
    - Mysteriousness
    - Curiosity
    - Comfort
    - Motivation
    - Laziness
    - None
    - Other [if any]

14. Select all emotions your child has experienced with the sight, or thought, of the color orange:

- Happiness
- Sadness
- Fear
- Anger
- Boredom
- Excitement
- Amusement
- Peace
- Love
- Hate
- Playfulness
- Safety
- Danger
- Mysteriousness
- Curiosity
- Comfort
- Motivation
- Laziness
- None
- Other [if any]

15. Select all emotions your child has experienced with the sight, or thought, of the color yellow:

- Happiness
- Sadness
- Fear
- Anger
- Boredom
- Excitement
- Amusement
- Peace
- Love
- Hate
- Playfulness
- Safety
- Trust
- Danger
- Mysteriousness
- Curiousness
- Comfort
- Motivation
- Laziness
- None
- Other [if any]

16. Select all emotions your child has experienced with the sight, or thought, of the color green:

- Happiness
- Sadness
- Fear
- Anger
- Boredom
- Excitement
- Amusement
- Peace
- Love
- Hate
- Playfulness
- Safety
- Trust
- Danger
- Mysteriousness
- Curiousness
- Comfort
- Motivation
- Laziness
- None
- Other [if any]

17. Select all emotions your child has experienced with the sight, or thought, of the color blue:

- Happiness
- Sadness
- Fear
- Anger
- Boredom
- Excitement
- Amusement
- Peace
- Love
- Hate
- Playfulness
- Safety
- Trust
- Danger
- Mysteriousness
- Curiousness
- Comfort
- Motivation
- Laziness
- None
- Other [if any]

18. Select all emotions your child has experienced with the sight, or thought, of the color purple:

- Happiness
- Sadness
- Fear
- Anger
- Boredom
- Excitement
- Amusement
- Peace
- Love
- Hate
- Playfulness
- Safety
- Trust
- Danger
- Mysteriousness
- Curiousness
- Comfort
- Motivation
- Laziness
- None
- Other [if any]

19. In the design of children's products (toys, clothes, articles), which red color do you prefer?

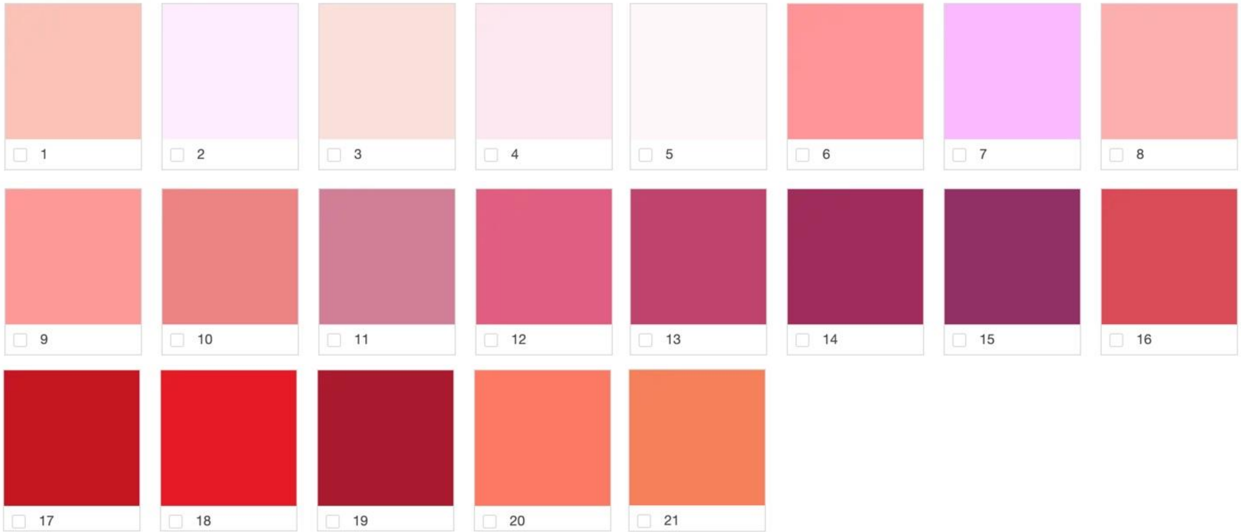

20. In the design of children's products (toys, clothes, articles), which orange color do you prefer?

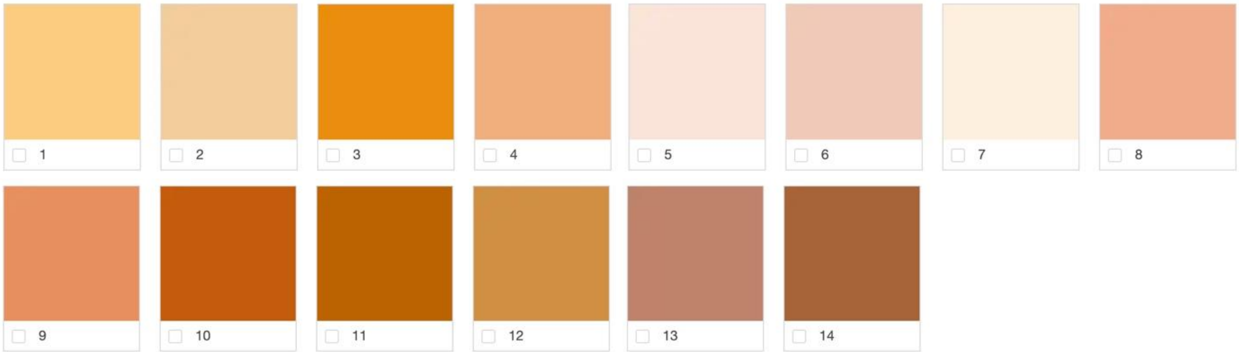

21. In the design of children's products (toys, clothes, articles), which yellow color do you prefer?

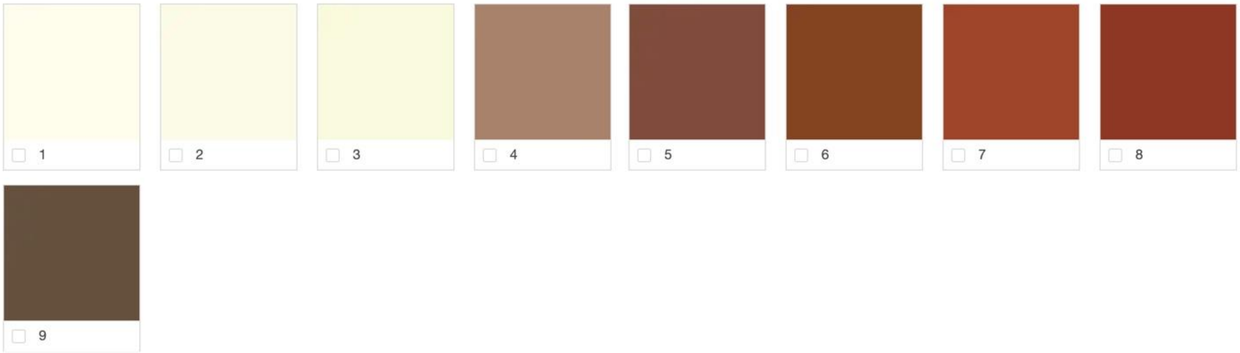

22. In the design of children's products (toys, clothes, articles), which green color do you prefer?

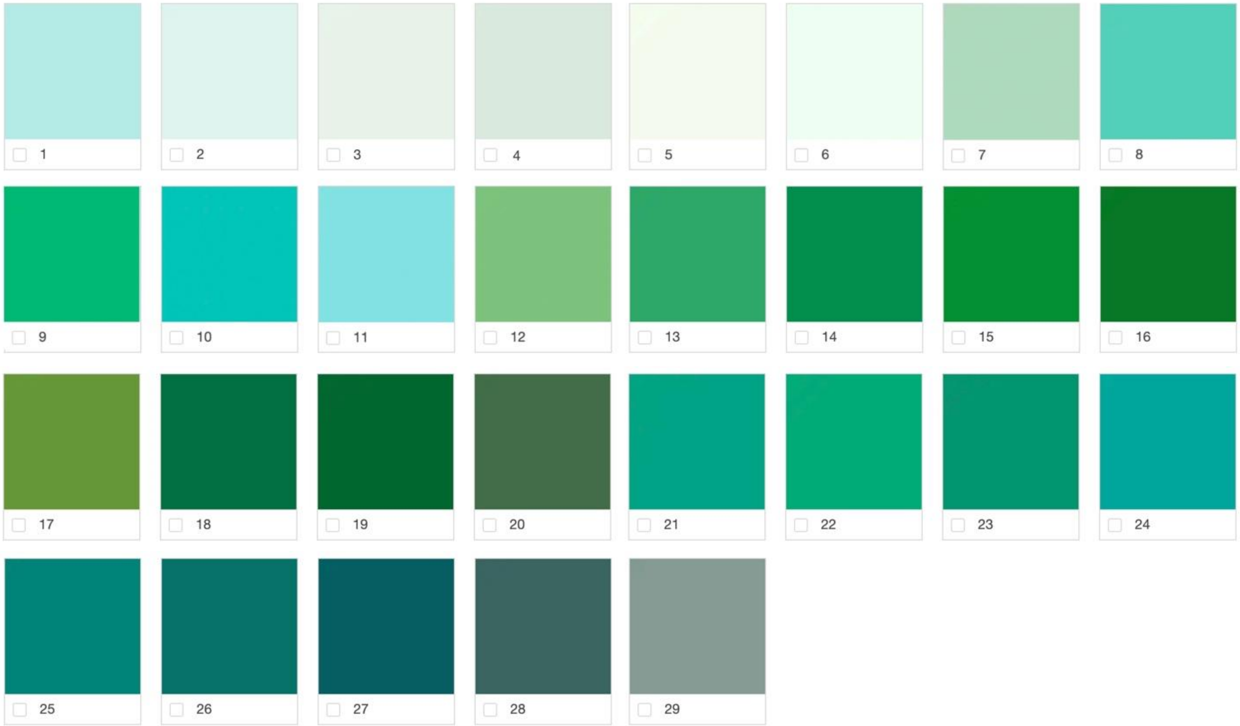

23. In the design of children's products (toys, clothes, articles), which blue color do you prefer?

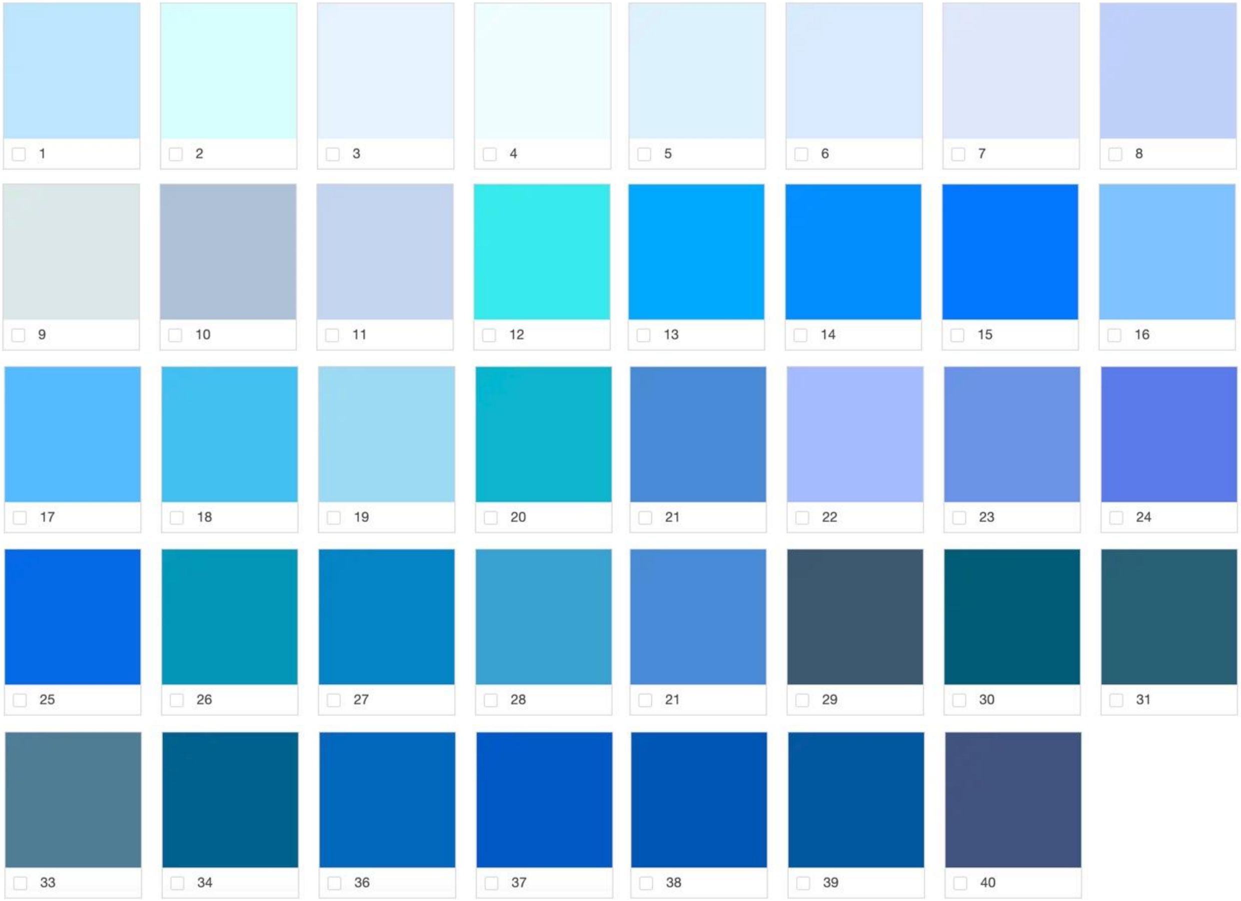

24. In the design of children's products (toys, clothes, articles), which blue color do you prefer?

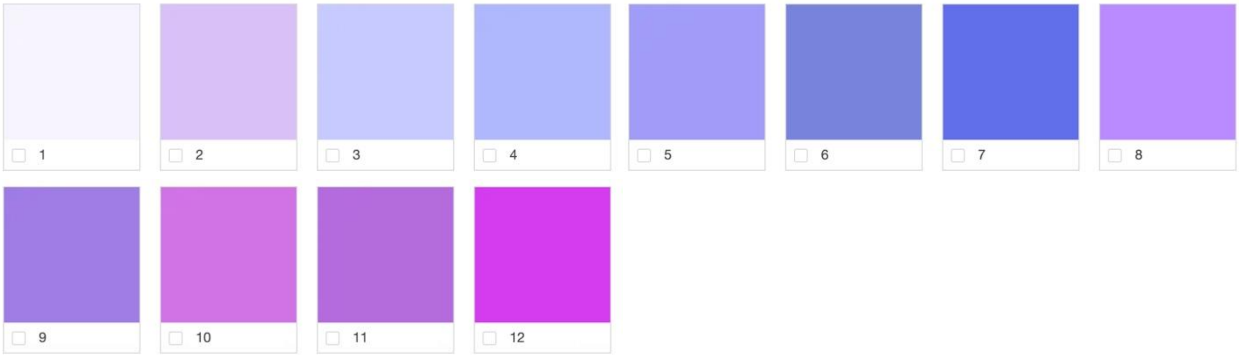

Survey 2

1. What is your gender?
    - Male
    - Female
2. What is your age?
    - 18~25
    - 25~35
    - 35~45
    - Above 45
3. Do you have children?
    - Yes
    - No
    - If you have no children, please stop the questionnaire and leave the page
4. What is the age of your child?
    - Under 1 year old
    - 2~5
    - 5~13
    - Above 13
5. What is your child's sex?
    - Male
    - Female
6. Please rate the following color scheme prepared for the design of children's products, according to your preferences:
    - Most preferred;
    - Preferred;
    - Neutral;
    - less preferred;
    - not preferred;

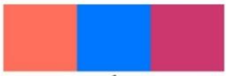

1

7. Please rate the following color scheme prepared for the design of children's products, according to your preferences:
    - Most preferred;
    - Preferred;
    - Neutral;
    - less preferred;
    - not preferred;

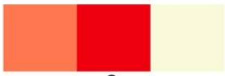

2

8. Please rate the following color scheme prepared for the design of children's products, according to your preferences:
    - Most preferred;
    - Preferred;
    - Neutral;
    - less preferred;
    - not preferred;

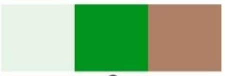

3

9. Please rate the following color scheme prepared for the design of children's products, according to your preferences:

- Most preferred;
- Preferred;
- Neutral;
- less preferred;
- not preferred;

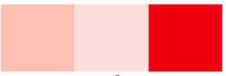

4

10. Please rate the following color scheme prepared for the design of children's products, according to your preferences:
    - Most preferred;
    - Preferred;
    - Neutral;
    - less preferred;
    - not preferred;

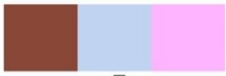

5

11. Please rate the following color scheme prepared for the design of children's products, according to your preferences:
    - Most preferred;
    - Preferred;
    - Neutral;
    - less preferred;
    - not preferred;

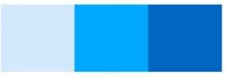

6

12. Please rate the following color scheme prepared for the design of children's products, according to your preferences:
    - Most preferred;
    - Preferred;
    - Neutral;
    - less preferred;
    - not preferred;

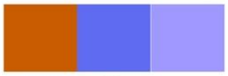

7

13. Please rate the following color scheme prepared for the design of children's products, according to your preferences:
    - Most preferred;
    - Preferred;
    - Neutral;
    - less preferred;
    - not preferred;

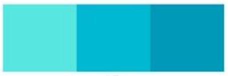

8

14. Please rate the following color scheme prepared for the design of children's products, according to your preferences:
    - Most preferred;
    - Preferred;
    - Neutral;
    - less preferred;
    - not preferred;

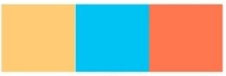

9

15. Please rate the following color scheme prepared for the design of children's products, according to your preferences:
    - Most preferred;
    - Preferred;

- Neutral;
- less preferred;
- not preferred;

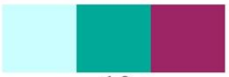

10

16. Please rate the following color scheme prepared for the design of children's products, according to your preferences:
    - Most preferred;
    - Preferred;
    - Neutral;
    - less preferred;
    - not preferred;

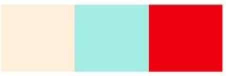

11

17. Please rate the following color scheme prepared for the design of children's products, according to your preferences:
    - Most preferred;
    - Preferred;
    - Neutral;
    - less preferred;
    - not preferred;

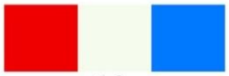

12

18. Please rate the following color scheme prepared for the design of children's products, according to your preferences:
    - Most preferred;
    - Preferred;
    - Neutral;
    - less preferred;
    - not preferred;

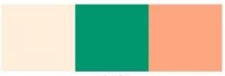

13

19. Please rate the following color scheme prepared for the design of children's products, according to your preferences:
    - Most preferred;
    - Preferred;
    - Neutral;
    - less preferred;
    - not preferred;

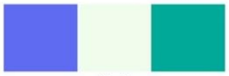

14

20. Please choose the appropriate adjective for the first color group:

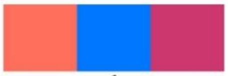

1

(a) casual
(b) dynamic and active
(c) heavy and deep
(d) graceful
(e) modern
(f) ethnic
(g) refreshing
(h) sporty
(i) striking
(j) fashionable

21. Please choose the appropriate adjective for the second color group:

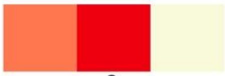

2

(a) decorative
(b) bright
(c) light
(d) cheerful
(e) young
(f) sharp
(g) dynamic and active
(h) mellow
(i) delicious
(j) emotional
(k) cute
(l) fresh
(m) sunny
(n) sporty
(o) sweet-sour
(p) rich
(q) colorful

22. Please choose the appropriate adjective for the third color group:

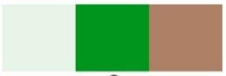

3

(a) quiet
(b) vivid
(c) mild
(d) generous
(e) tropical
(f) simple
(g) restful
(h) rustic
(i) conservative
(j) calm
(k) lively
(l) formal

23. Please choose the appropriate adjective for the 4th color group:

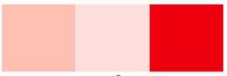

4

(a) tender
(b) calm
(c) casual
(d) childlike
(e) delicious
(f) soft
(g) sunny
(h) mild
(i) feminine
(j) cute
(k) innocent
(l) romantic
(m) mellow

24. Please choose the appropriate adjective for the 5th color group:

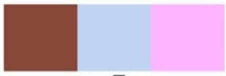

5

(a) vivid
(b) casual
(c) metallic
(d) mysterious
(e) chic
(f) interesting
(g) robust
(h) pretty
(i) fashionable

25. Please choose the appropriate adjective for the 6th color group:

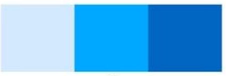

6

(a) soft
(b) active
(c) agile
(d) graceful
(e) calm
(f) refreshing
(g) healthy
(h) natural
(i) peaceful
(j) plain
(k) quiet
(l) pure
(m) clean

26. Please choose the appropriate adjective for the 7th color group:

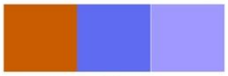

7

(a) conservative
(b) urban
(c) mysterious
(d) heavy and deep
(e) dreamy
(f) alluring

27. Please choose the appropriate adjective for the 8th color group:

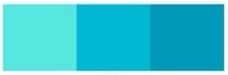

8

(a) clean and fresh
(b) emotional
(c) light
(d) lively
(e) youthful
(f) natural
(g) open
(h) peaceful
(i) healthy
(j) innocent
(k) calm
(l) refreshing
(m) pure
(n) clean

28. Please choose the appropriate adjective for the 9th color group:

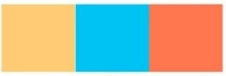

9

(a) sunny
(b) vigorous-active
(c) vivid
(d) sweet-sour
(e) sporty
(f) bright
(g) dynamic
(h) fascinating
(i) agile
(j) friendly
(k) amusing
(l) childlike
(m) colorful
(n) happy
(o) pleasant

29. Please choose the appropriate adjective for the 10th color group:

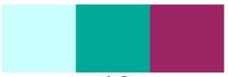

10

(a) agile
(b) cultural
(c) metallic
(d) neat
(e) modest
(f) pure
(g) stylish
(h) light
(i) calm
(j) healthy
(k) peaceful
(l) quiet

30. Please choose the appropriate adjective for the 11th color group:

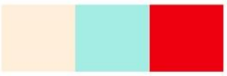

11

(a) alluring
(b) sporty
(c) fresh and young
(d) pretty
(e) striking
(f) amiable
(g) friendly
(h) pleasant
(i) mellow

31. Please choose the appropriate adjective for the 12th color group:

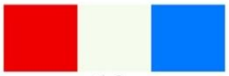

12

(a) sporty
(b) active and vigorous
(c) decorative
(d) fashionable
(e) vivid
(f) colorful
(g) bright
(h) agile
(i) modern
(j) ethnic
(k) sharp
(l) pretty

32. Please choose the appropriate adjective for the 13th color group:

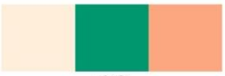

13

(a) mild
(b) natural
(c) tropical
(d) soft
(e) quiet
(f) pure
(g) restful
(h) fresh
(i) neat
(j) open
(k) light
(l) lively
(m) calm

33. Please choose the appropriate adjective for the 14th color group:

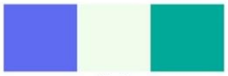

14

(a) quiet
(b) natural
(c) youthful
(d) lively
(e) light
(f) pure
(g) healthy
(h) innocent
(i) friendly
(j) clean and fresh
(k) neat
(l) peaceful
(m) free
